# Knowledge, attitudes, and practice on the prevention of central line-associated bloodstream infections among nurses in oncological care: A cross-sectional study in an area of southern Italy

**DOI:** 10.1371/journal.pone.0180473

**Published:** 2017-06-30

**Authors:** Maria Rosaria Esposito, Assunta Guillari, Italo Francesco Angelillo

**Affiliations:** 1 Istituto Nazionale Tumori, “Fondazione G. Pascale”, Naples, Italy; 2 Department of Experimental Medicine, University of Campania “Luigi Vanvitelli”, Naples, Italy; Universita degli Studi di Ferrara, ITALY

## Abstract

The objectives of the cross-sectional study were to delineate the knowledge, attitudes, and behavior among nurses regarding the prevention of central line-associated bloodstream infections (CLABSIs) and to identify their predisposing factors. A questionnaire was self-administered from September to November 2011 to nurses in oncology and outpatient chemotherapy units in 16 teaching and non-teaching public and private hospitals in the Campania region (Italy). The questionnaire gathered information on demographic and occupational characteristics; knowledge about evidence-based practices for the prevention of CLABSIs; attitudes towards guidelines, the risk of transmitting infections, and hand-washing when using central venous catheter (CVC); practices about catheter site care; and sources of information. The vast majority of the 335 nurses answered questions correctly about the main recommendations to prevent CLABSIs (use sterile gauze or sterile transparent semipermeable dressing to cover the catheter site, disinfect the needleless connectors before administer medication or fluid, disinfect with hydrogen peroxide the catheter insertion site, and use routinely anticoagulants solutions). Nurses aged 36 to 50 years were less likely to know these main recommendations to prevent CLABSIs, whereas this knowledge was higher in those who have received information about the prevention of these infections from courses. Nurses with lower education and those who do not know two of the main recommendations on the site’s care to prevent the CLABSIs, were more likely to perceive the risk of transmitting an infection. Higher education, attitude toward the utility allow to dry antiseptic, and the need of washing hands before wearing gloves for access to port infusion were predictors of performing skin antiseptic and aseptic technique for dressing the catheter insertion site. Educational interventions should be implemented to address the gaps regarding knowledge and practice regarding the prevention of CLABSIs and to ensure that nurses use evidence-based prevention interventions.

## Introduction

The issue of Health Care-Associated Infections (HAIs) continues to be one of the most important public health problems in many countries throughout the world [1,2], and these infections remain a leading cause of morbidity and mortality among hospitalized patients [3] also with an increase consumption of resources and add to cost [4,5]. Central line-associated bloodstream infections (CLABSIs), the majority related with the use of the central venous catheter (CVC) [6,7], are the most important complications in critical care [8–10] and in cancer settings [11,12]. In particular, in cancer settings, several risk factors have been identified for CLABSIs in patients who undergo chemotherapy such as, for example, a prolonged duration of catheterization, microbial colonization at the insertion site and of the catheter hub, and inadequate care/maintenance of the CVC after insertion [13–15]. Therefore, improving the quality of the health care is a key priority, since reduction of CLABSIs may be achieved through efforts with adherence to appropriate preventive measures in line with evidence-based recommendations [16,17].

Clinical practice guidelines have been published by several international organisms for the prevention of CLABSIs [18–21] that generally include specific actions to be implemented by health care workers (HCWs) who insert and handle a CVC. Among the HCWs, nurses in cancer settings have the most direct and continuous role in performing high-risk CVC procedures and they should be knowledgeable and compliant in the insertion assistance, care, and maintenance of central lines. Therefore, they are well positioned to implement the recommendations and have a unique opportunity to contribute to primary prevention of these infections via evidence-based best practices [22]. Information has been reported in several surveys in different countries regarding the level of knowledge, the attitudes, and the extent to which evidence-based practices are used among the nurses in different setting [23–29]. To improve the appropriate use of CVCs, in-depth knowledge of the issues by nurses is essential and obtaining this information will contribute to develop preventive programs in order to reduce the frequency of CLABSIs in the cancer setting. Therefore, the objective of the present investigation was to delineate the level of knowledge, attitudes, and behavior regarding CVC procedures among a large sample of nurses working in cancer setting in the Italian hospitals. Furthermore, the second objective was to identify the predisposing factors for the knowledge, attitudes, and behavior.

## Materials and methods

The study had a cross-sectional design and was carried out in all 16 non-teaching and teaching public and private hospitals whose units utilized CVCs for oncological patients in the geographic area of Avellino (Ariano Irpino, San Giuseppe Moscati), Benevento (Gaetano Rummo), Caserta (Santa Anna and San Sebastiano), Naples (Cardarelli, Evangelico Villa Betania, Istituto Nazionale Tumori, Cotugno, Monaldi, San Gennaro, Santa Maria della Pietà, Santa Maria delle Grazie, Santobono/Pausillipon, Second University Teaching Hospital, University Federico II Teaching Hospital), and Salerno (San Giovanni di Dio and Ruggi d’Aragona) (Italy).

Recruitment took place from September to November 2011. The target group was all 472 nurses working in oncology and outpatient chemotherapy units, involved in the care of patients with CVC, in the selected hospitals.

The head physician of each hospital was invited to participate with a letter, they were also informed about the voluntary and anonymous participation and the study coordinator, so that they could contact for ask questions, and questionnaires with detailed instructions about the modality of gather the information. Participants were informed about the purposes of the study, that the data would be handled confidentially, and that the results would be reported at an aggregated group rather than at an individual level. Written informed consent was obtained before the return of the questionnaire. The questionnaire did not contain identifying information and it was self-administered to the sample that agreed to participate. No incentives or financial payments have been provided for the participation in the study.

The questionnaire was developed by the research team, based on the CDC Guidelines for the Prevention of Intravascular Catheter Related Infections [18]. The complete questionnaire in Italian and English are found in S1 Questionnaire and S2 Questionnaire. The questions were distributed through five parts. The first part recorded data on nurses’ demographic and professional characteristics (age, gender, educational level, years of working activity, unit of activity). The second part was designed to assess nurses’ knowledge about evidence-based practices for the prevention of CLABSIs, such as frequency of CVC dressing changes, use of sterile gauze or transparent semipermeable dressings to cover the catheter site, antiseptic agent to use in skin for catheter insertion site, use of topical antibiotic ointment at CVC insertion site, flush the lumen after the administration of medication or fluid, replacement of intravenous (IV) administration sets, disinfect IV ports/needleless connectors before accessing, and use routinely anticoagulants solutions. The options of the responses were “yes”, “do not know”, and “no”. The third part was composed on questions measuring awareness towards the utility of guidelines, perception of the risk of transmitting a CLABSI when handling the CVC and necessity of hand hygiene before and after dressing changes of the catheter site, monitor the catheter site visually when changing the dressing or by palpation through an intact dressing on a regular basis, and hands hygiene before wear gloves for access to port infusion. Three questions were scored using 3-point Likert-type scale with options for “agree”, “uncertain”, and “disagree” and three questions using a 10-point Likert-type scale, ranging from 1 to 10. The fourth part collected information on practices about catheter site care, dressing and aseptic technique for dressing changes of the catheter insertion site, aseptic technique and correct frequency about disinfection of IV access ports and needleless connectors before accessing or manipulation, replacement of IV administration sets, and replacement sets used to administer blood, blood product, and lipid emulsions. The questions were measured using a 5-point Likert-type scale ranging from “never” to “always”, and closed-end questions investigated nurses’ practice toward the prevention of CLABSIs. The last part queried nurses on their source of information about CLABSIs and about their interest in acquiring new information about prevention of CLABSIs.

The questionnaire was pilot tested with a group of 20 oncology nurses in order to check the clarity and readability of the questionnaire. The final version of the questionnaire was refined and corrected based on feedback from the participants.

Prior to commencement of data collection, ethical approval for the conduction of the research was granted from the Ethics Committee of the Second University of Naples.

### Statistical analysis

A univariate analysis was performed with a *t*-test or chi-square test as appropriate in order to assess the significant association between outcomes variables and the explanatory variables. Variables with *p*-value ≤ 0.25 on univariate analysis were considered for possible entry in the multivariate linear and logistic regressions models. Three separate multivariate stepwise logistic and linear regression models were constructed for identification of the independent association with the selected predictors and the following outcomes of interest: knowledge about the main recommendations to prevent CLABSIs (Model 1); perception of the risk of transmitting a CLABSI when handling the CVC (Model 2); and appropriate behavior about skin antiseptic and aseptic technique for dressing the catheter insertion site (Model 3). In Model 1, nurses were divided in those who knew about the use sterile gauze or sterile transparent semipermeable dressing to cover the catheter site, disinfect the needleless connectors before administer medication or fluid, disinfect with hydrogen peroxide the catheter insertion site, and use routinely anticoagulants solutions versus all others, and in Model 3, nurses were divided in those who had an appropriate behavior about skin antiseptic and aseptic technique for dressing the catheter insertion site versus all others.

The following explanatory variables were included in all models: gender (male = 0; female = 1), age (categories: ≤35 = 1; 36–40 = 2; 41–45 = 3; 46–50 = 4; >50 = 5), educational level in Nursing (Regional Diploma = 0; University Diploma/Degree = 1), number of years in practice (1–5 = 1; 6–10 = 2; 11–15 = 3; 16–20 = 4; >20 = 5), work setting (outpatient chemotherapy unit = 0; oncology ward = 1), workshops and courses as sources of information on the prevention of CLABSIs (no = 0; yes = 1), and need of additional information about the prevention of CLABSIs (no = 0; yes = 1). The following variables were also included: knowledge about the guidelines for the preventions of CLABSIs regarding dressing changes of catheter insertion site (no = 0; yes = 1), skin antiseptic preparations used for dressing replacement (no = 0; yes = 1), routinely use anticoagulant therapy to reduce the risk of CLABSIs (no = 0; yes = 1), and disinfection of IV ports/needleless connectors before access port (no = 0; yes = 1) in Model 1; perceived the risk of transmitting a CLABSI (continuous) in Model 2; appropriate behavior about skin antiseptic and aseptic technique for dressing the catheter insertion site (no = 0; yes = 1), positive attitude toward the utility of allow to dry antiseptic (no = 0; yes = 1), and necessity of hands hygiene before wearing gloves for access to port infusion (no = 0; yes = 1) in Model 3; correct knowledge about the use of the sterile gauze or transparent semipermeable dressing to cover catheter site and change the dressing of the insertion site every 7 days for semipermeable dressing or if the dressing is soiled or loosened (no = 0; yes = 1) in Models 2 and 3.

The stepwise selection procedure with a forward method was used, and a significance level of 0.2 was used as the criterion for variables to enter in the regression models and 0.4 for variables to remain. Results of the logistic regression models are presented as odds ratios (ORs) with 95% confidence intervals (CIs) and *p*-values. Results of the linear regression model are presented as standardized regression coefficients (β). *P*-values of ≤0.05 were considered statistically significant, and all analyses were two-sided. All data (S3 Data Set) were analyzed using the statistical software Stata 10.1.

## Results

### Demographic and professional characteristics

A total of 335 questionnaires were collected back out of 472 that were initially distributed, giving a response rate of 71%. The socio-demographic and professional characteristics of respondents are shown in Table 1. Approximately two-thirds were female (61.3%) with a mean age of 43 years, the vast majority was in oncology wards, and the average number of years working in these wards was 9.9.

**Table 1:** Main socio-demographic characteristics of the responders.

	n*	%
*Gender*		
Female	203	61.3
Male	128	38.7
*Age group*, *(years)*		42.9±8.9 (25–65)^**+**^
≤35	70	21
36–40	72	21.7
41–45	69	20.8
46–50	57	17.2
>50	64	19.3
*Number of years in practice*		9.9±8.7 (1–38)^**+**^
1–5	131	39.5
6–10	98	29.5
11–15	22	6.6
16–20	49	14.8
>20	32	9.6
*Educational level in Nursing*		
Regional Diploma	236	70.5
University Diploma/Degree	99	29.5
*Professional role*		
Ordinary nurse	310	94.5
Head nurse	18	5.5
*Work setting*		
Oncology ward	284	84.8
Outpatient chemotherapy unit	51	15.2

* Number of responding

^**+**^ Mean±Standard deviation (range)

### Knowledge about evidence-based practice for the prevention of CLABSIs

The survey responses related to knowledge regarding evidence-based practices for the prevention of CLABSIs are reported in Table 2. The vast majority of nurses, with frequencies ranging from 70.7% to 90.1%, answered questions correctly about to flush the lumen with saline after administration of medication or fluid, to use sterile gauze or transparent semipermeable dressing to cover the catheter site, to not use topical antibiotic ointment on insertion site, and to replace IV administration sets every 72 hours. Only 64.4% acknowledged that the routinely use of anticoagulants solutions does not prevent CLABSIs and 70% that hydrogen peroxide is not recommended to disinfect the catheter insertion site.

**Table 2:** Knowledge about evidence-based practices for the prevention of CLABSIs.

	Yes	No	Do not know
	(%)	(%)	(%)
Flush the lumen with normal saline after the administration of medication or fluid	**90.1**	7.2	2.7
Use sterile gauze or sterile transparent semipermeable dressing to cover the catheter site	**85.9**	9.3	4.8
Disinfect the needleless connectors before administer medication or fluid	**77.5**	12.3	10.2
Replace catheter site dressing every 7 days or if the dressing becomes visibly soiled or loosened	**76**	19.5	4.5
Use topical antibiotic ointment on catheter insertion site	16.5	**75.5**	8
Replace the IV administration sets every 72 hours	**70.7**	19.7	9.6
Disinfect with hydrogen peroxide the catheter insertion site	20	**70**	10
Use routinely anticoagulants solutions	26	**64.4**	9.6

In bold are indicated the correct answers

The results from the multivariate logistic and linear regression models exploring the association between the different variables and the outcomes of interest are shown in Table 3. Nurses in the age groups from 36–40 years (OR = 0.35; 95% CI 0.16–0.76), 41–45 (OR = 0.41; 95% CI 0.19–0.88), and 46–50 (OR = 0.4; 95% CI 0.17–0.94), when the age ≤35 years was chosen as reference category, were less likely to know the main recommendations to prevent CLABSIs, whereas this knowledge was higher in those who have received information about the prevention of CLABSIs from workshops and courses (OR = 2.44; 95% CI 1.29–4.62) (Model 1).

**Table 3:** Multivariate logistic (1 and 3) and linear (2) regression models results.

Variable	OR	95% CI	*p*
**Model 1**. Knowledge about the main recommendations to prevent CLABSIs			
Log likelihood = -155.08, chi-square = 21.04 (5 df), *p* = 0.0008			
Workshops and courses as sources of information about prevention of CLABSIs	2.44	1.29–4.62	0.006
Age			
≤35*	1.0	-	-
36–40	0.35	0.16–0.76	0.008
41–45	0.41	0.19–0.89	0.023
46–50	0.4	0.17–0.94	0.035
Need of additional information about the prevention of CLABSIs	1.75	0.73–4.22	0.21
**Model 3**. Appropriate behavior about skin antiseptic and aseptic technique for dressing the catheter insertion site			
Log likelihood = -164.46, chi-square = 87.08 (7 df), *p*< 0.00001			
Attitude toward the utility allow to dry antiseptic	7.31	3.96–13.49	<0.001
Attitudes toward the necessity of hands hygiene before wearing gloves for access to port infusion	11.85	3.43–40.89	<0.001
Educational level in Nursing	2.11	1.17–3.8	0.012
Age			
≤35*	1.0	-	-
41–45	1.41	0.7–2.84	0.33
46–50	0.67	0.33–1.36	0.27
Workshops and courses as sources of information about prevention of CLABSIs	1.43	0.82–2.49	0.21
Knowledge about the use of the sterile gauze or transparent semipermeable dressing to cover catheter site and change the dressing of the insertion site every 7 days or if the dressing is soiled or loosened	1.32	0.71–2.44	0.38
**Variable**	**Coeff.**	***t***	***p***
**Model 2.** Perception of the risk of transmitting a CLABSI			
F (2,328) = 7.25, R^2^ = 4.2%, adjusted R^2^ = 3.7%, *p* = 0.0008			
Knowledge about the use of the sterile gauze or transparent semipermeable dressing to cover catheter site and change the dressing of the insertion site every 7 days or if the dressing is soiled or loosened	-0.85	-2.66	0.008
Educational level in Nursing	-0.85	-2.6	0.01
Constant	7.15	-	-

*Reference category

### Attitudes towards the prevention of CLABSIs

Attitudes towards the effectiveness of guidelines and of hand hygiene before and after dressing changes of catheter insertion site, on a scale from 1 to 10, showed a mean score for the whole sample respectively of 9.1±1.5 and 9.7±1. Respondents indicated their perceived risk of transmitting to a patient a CLABSI when handling the CVC, on a scale from 1 to 10, with a mean score of 6.3±2.8. Moreover, 81.8% agreed with recommendations about monitoring the catheter site visually or by palpation through an intact dressing on a regular basis, 85.3% disagree that the use of gloves before the infusion port access replaces the need of hands washing, and only 55.2% agreed that is useful to dry the antiseptic on the insertion site before catheter insertion. The multivariate linear regression analysis allowed to identify several variables associated with the dependent variable “perception of the risk of transmitting a CLABSI”. Nurses with a lower level of education and those who do not know two of the main recommendations on the site’s care for the prevention of catheter related infections were more likely to perceive the risk (Model 2 in Table 3).

### Practices about the prevention of CLABSIs

Among the different activities related to the care of patients with CVC, the vast majority was involved in catheter site care. Appropriate procedures were self-reported regarding hands hygiene before dressing (84.6%), hands hygiene with antiseptic soap (81.7%), hands washing for more than 1 minute (58%), use of gloves during dressing replacement (93.8%), povidone-iodine for dressing insertion sites (87.8%), and let the antiseptic dry (33.3%). Results of the multivariate logistic regression model carried out with the practices regarding skin antiseptic and aseptic technique for dressing of the insertion catheter site as outcome variable, revealed that nurses with a graduate degree were 2.1 times more likely to have an appropriate behavior than those with a lower education (95% CI 1.17–3.79). Respondents with a better attitude toward the utility allow to dry antiseptic and toward the need of washing hands before wearing gloves for access to port infusion were respectively 7.31 (95% CI 3.96–13.49) and 11.85 (95% CI 3.43–40.88) more likely to perform skin antiseptic and aseptic technique for dressing the catheter insertion site (Model 3 in Table 3). The responses related to the disinfection of IV access ports indicated that 75.3% of the sample self-reported always disinfects the port infusion with povidone iodine (72.8%). A total of 73% and 79% always wash their hands and wear gloves before replacement of IV administrations sets, respectively. Only 60% replace the sets within 24 hours of initiating the infusion with lipid emulsions, 76.9% always disinfect IV access port needleless connectors before accessing or manipulation, but only 27% swabbing with chlorhexidine 2% as antiseptic agent to clean the access hub or connector, and 76.7% always flushed the lumens with normal saline after IV therapy.

Nurses indicated workshops and courses (67.3%) as their main source of information about the prevention of CLABSIs, followed by guidelines (42.7%), and internet (30.7%). The vast majority (83.7%) reported that they would like to learn more.

## Discussion

The data of the current study are among the first reporting knowledge, attitudes, and evidence-based practices for the prevention of CLABSIs among a large sample of nurses working in oncology settings in Italy.

This study demonstrated that nurses have an adequate level of knowledge concerning evidence-based recommendations for preventing CLABSIs, since the vast majority were aware about the main recommendations for patients with CVCs, such as the type of dressing (85.9%) and/or frequency changes (76%) of the catheter insertion site. These findings are comparatively higher than the values observed in nurses in the adult ICU with knowledge of 10% [30] and of 43.4% and 26.2% [26], in ICU and outside ICUs with 37.4% and 62% [27], in ICUs and other wards with 63.9% [24], and in neonatal and pediatric ICUs with 16.7% and 71.8% [31]. In contrast, there are wide areas where the knowledge was lower, particularly regarding the agent for the skin antisepsis, since 30% did not know that hydrogen peroxide is not indicated. These findings suggest the need for including the current evidence-based practice guidelines in educational curricula and programs for HCWs to help them in improving their knowledge. This is also supported by the fact that over three-fourths of the nurses reported that they would like to learn more about the prevention of CLABSIs. Moreover, provision of information about the guidelines for the prevention of CLABSIs influences knowledge since nurses were able to answer correctly if they have received information from workshops and courses.

The findings from this survey showed that respondents had an extremely positive attitude towards the utility of guidelines for the prevention of CLABSIs. A similar result has been observed in nurses in surgical wards in Italy about the utility of guidelines and protocols for disinfection procedures for the prevention of HAIs [32]. Positive attitudes were also recorded regarding the necessity of washing hands before and after dressing the insertion site. The multivariable regression analysis showed that nurses with a high level of perceived risk of transmitting a CLABSI were those with a Regional Diploma level of education and who did not know the recommendations for the care of the catheter site.

Although most nurses’ claims that guidelines were necessary in order to reduce CLABSIs, hands hygiene is alarmingly low and with the current evidence that the hands represent the main route of transmission of hospital pathogens, hands hygiene is one of the priority measures on health programs and actions destined to prevent infections. Low adherence to hand hygiene (40–72%) on the nurses’ practice toward CVC’s care has been reported in teaching and non-teaching hospital ICUs in Yemen [33] and only 26% of the sample that washed their hands successful in Mongolia [34]. Before central venous catheter insertion, only 22.5% of nurses performed hand hygiene in the ICUs in Egypt [30]. Regarding the use of chlorhexidine 2%, only 27% of the sample used it as antiseptic agent to clean the access port or needleless connectors. This is in accordance with similar previous studies [35, 36].

Based on multivariate regression analysis, the knowledge of recommendations on dressing insertion site was significantly associated with the perception of the risk of transmitting a CLABSI when handling the CVC. Moreover, workshops and courses as source of information were significantly associated with a higher level of knowledge about the main recommendations for the prevention of CLABSIs. The importance of continuous training process that involves nurses allows them to acquire new skills for the CVCs management can explain this finding. Furthermore, the analysis of the predictors of the knowledge also showed that those younger were more knowledgeable. A possible explanation is that nurses of younger age are more likely to take advantage from educational level in university compared to those older with different educational path. This is confirmed by the finding that nurses with a graduate degree perform an appropriate behavior about skin antiseptic and aseptic technique for dressing the catheter insertion site.

Cautions needs to be taken while interpreting this data from the questionnaire as some potential limitations associated with the design and measurements need to be addressed. First, as this is a cross-sectional study, any causative relationship between the examined variables and the outcomes of interest was difficult to determine. Second, similar to all data that are based on self-report questionnaires, the accuracy of the results was heavily dependent on the honesty and understanding of the respondents, there could have been potential for recall bias. Third, as a general limitation to questionnaire, nurses may tend to provide more socially desirable responses which may show appropriate practice but may not reflect reality. To overcome social desirability bias and improve the validity of the data, it has been used an anonymous self-administered questionnaire which may minimise social desirability bias and increase respondents’ willingness to participate. Fourth, the study was limited to nurses in oncology units, so the results may not be generalizable to other nurses and HCW populations, although this was not an aim of the survey. In spite of these limitations, the large sample size and the high response rate in the study reduce the likelihood of bias in the sample and this study provides primary yet valuable data.

## Conclusions

In conclusion, the results of this study clearly indicated that the educational interventions are very important and should be implemented to address the gaps regarding knowledge and practice and to ensure that nurses use evidence-based prevention interventions.

## Supporting information

S1 Questionnaire (DOC)

S2 Questionnaire (DOC)

S1 Dataset (XLS)
