# Supplementary material for: Knowledge, attitudes, and practice on the prevention of central line-associated bloodstream infections among nurses in oncological care: A cross-sectional study in an area of southern Italy
Source: PLoS One. 2017 Jun 30;12(6):e0180473. doi: 10.1371/journal.pone.0180473 (PMC5493401; doi:10.1371/journal.pone.0180473)
Supplement: S1 Questionnaire — (DOC) [file pone.0180473.s001.doc]

**QUESTIONARIO**

1. **CARATTERISTICHE SOCIO-ANAGRAFICHE E PROFESSIONALI**

**Vorrei raccogliere informazioni sulle Sue caratteristiche socio-anagrafiche e professionali.**

**A1**. Qual è il Suo genere?  Maschile  Femminile **A2**. Quanti anni ha compiuto l’ultimo compleanno? **_______**

**A3**. Qual è il titolo di studio più elevato che ha conseguito?

 Diploma regionale in Infermieristica  Diploma Universitario/Laurea in Infermieristica

**A4.** Da quanti anni lavora in un reparto di oncologia? **________**

**A5**. Qual è il Suo ruolo professionale?  Infermiere  Caposala/Coordinatore Infermieristico

**A6**. Dove svolge la Sua attività?  Unità operativa con degenza  Ambulatorio di chemioterapia  Altro**________________**

1. **CONOSCENZE**

**Vorrei esplorare le Sue conoscenze sulle batteriemie correlate al Catetere Venoso Centrale (CVC).**

| *Le Linee Guida per la pratica clinica sono documenti che includono raccomandazioni finalizzate a ottimizzare l'assistenza al paziente fondate su una revisione sistematica delle prove di efficacia e su una valutazione di benefici e danni di opzioni assistenziali alternative al fine di aiutare gli operatori sanitari ed i pazienti* |
| --- |

**B1.** Le Linee Guida sono necessarie al fine di **(è possibile più di una risposta)**:

**1.1**  ridurre la variabilità nella pratica clinica **1.2**  prendere decisioni basate sulle migliori evidenze scientifiche disponibili

**1.3**  ridurre i rischi per i pazienti **1.4**  fornire pratiche assistenziali appropriate e sicure per i pazienti

**1.5**  promuovere un uso efficiente delle risorse

| **B2. Quali misure sono raccomandate per la prevenzione delle batteriemie correlate al CVC?** | Si | No | Non so |
| --- | --- | --- | --- |
| **2.1** Lavare il lume con soluzione fisiologica dopo la somministrazione dei farmaci o fluidi |  |  |  |
| **2.2** Utilizzare una garza o medicazione sterile semipermeabile trasparente per coprire il sito di inserimento del catetere |  |  |  |
| **2.3** Disinfettare i connettori senza ago prima di somministrare farmaci o fluidi |  |  |  |
| **2.4** Sostituire la medicazione sterile semipermeabile trasparente del sito del catetere ogni 7 giorni o la garza quando è visibilmente sporca o è staccata |  |  |  |
| **2.5** Utilizzare una pomata antibiotica sul sito di inserimento del catetere |  |  |  |
| **2.6** Sostituire il set di infusione ogni 72 ore dall’inizio della somministrazione di liquidi |  |  |  |
| **2.7** Disinfettare con perossido di idrogeno il sito di inserimento del catetere |  |  |  |
| **2.8** Utilizzare soluzioni anticoagulanti di routine |  |  |  |

1. **ATTITUDINI**

**Vorrei conoscere i Suoi atteggiamenti nei confronti delle batteriemie correlate al CVC. Risponda alle seguenti domande nel modo più sincero possibile.**

**C1.** Su una scala da 1 a 10, quanto pensa siano utili le Linee Guida per la prevenzione delle batteriemie correlate al CVC, dove 1 indica nessuna utilità e 10 elevata utilità

1 2 3 4 5 6 7 8 9 10

**C2.** Su una scala da 1 a 10, quanto si sente a rischio di trasmettere una batteriemia correlata al CVC durante la manipolazione, dove 1 indica nessun rischio e 10 alto rischio

1 2 3 4 5 6 7 8 9 10

**C3.** Su una scala da 1 a 10, quanto ritiene utile per la riduzione delle batteriemie correlate al CVC l’igiene delle mani prima e dopo la sostituzione della medicazione, dove 1 indica nessuna necessità e 10 elevata necessità

1 2 3 4 5 6 7 8 9 10

| **Per le seguenti procedure finalizzate alla prevenzione delle batteriemie correlate al CVC risponda se è d’accordo, incerto o disaccordo** | **D’accordo** | **Incerto** | **Disaccordo** |
| --- | --- | --- | --- |
| **C.4** Monitorare regolarmente il sito di inserimento del catetere attraverso l’ispezione visiva o la palpazione mantenendo integra la medicazione |  |  |  |
| **C.5** Lasciare asciugare l’antisettico sul sito di inserzione prima dell’inserimento del catetere |  |  |  |
| **C.6** Utilizzare i guanti prima di accedere alla porta di infusione sostituisce il lavaggio  delle mani |  |  |  |

1. **COMPORTAMENTI**

**Vorrei raccogliere informazioni sul Suo comportamento.**

**D1**. **Quali delle seguenti azioni esegue nella gestione dei CVC?**

| 1. **Sostituisce la medicazione sul sito di inserzione del CVC?**   Si  No **(andare alla domanda 2)**    1. Con quale frequenza lava le mani prima di sostituire la medicazione del sito del CVC?    mai **(andare alla domanda 1.3)**  raramente  talvolta  spesso  sempre   - 1. Che cosa utilizza per lavare le mani?  antisettico  sapone  altro, specificare **___________**   **1.2.1** Per quanto tempo lava le mani? **_________________**   - 1. Con quale frequenza indossa i guanti prima di sostituire la medicazione del sito del CVC?    mai **(andare alla domanda 1.4)**  raramente  talvolta  spesso  sempre  **1.3.1** Quale tipo di guanti utilizza?  non-sterili   inizialmente non-sterili per rimuovere la vecchia medicazione, poi sterili per medicare   non-sterili con tecnica “no touch”   sterili  **1.4** Dopo quanti giorni sostituisce la medicazione? ________  **1.5** Che cosa utilizza per la preparazione dell’antisepsi della cute?  clorexidina al 2%  iodopovidone  altro, specificare ___________  **1.6** Prima della disinfezione, pulisce il sito di inserimento del CVC?  No **(andare alla domanda 2)**   Si, con soluzione fisiologica  Si, con altro________________  **1.7** Lascia asciugare l’antisettico prima di procedere alla copertura del sito?   No **(andare alla domanda 2)**   Si **1.7.1** Per quanto tempo? ______ |
| --- |
| 1. **Esegue la disinfezione della porta di accesso del CVC prima di somministrare la terapia?**    mai **(andare alla domanda 3)**  raramente  talvolta  spesso  sempre  **2.1** Che cosa utilizza per la disinfezione della porta di accesso del CVC?  clorexidina al 2%  iodopovidone  altro, specificare ________________  **2.2** Lascia asciugare l’antisettico prima di procedere?   No  **(andare alla domanda 3)**   Si **2.2.1** Per quanto tempo? ______ |
| **3. Sostituisce i set di infusione?**  Si  No **(andare alla domanda 4)**  **3.1** Lava le mani prima di sostituire i set di infusione?   mai  raramente  talvolta  spesso  sempre  **3.2** Indossa i guanti prima di sostituire i set di infusione?   mai  raramente  talvolta  spesso  sempre  **3.3** Dopo quante ore dalla somministrazione di emulsioni lipidiche, sostituisce i set di infusione? **_________**  **3.4** Dopo quante ore dalla somministrazione di sangue ed emoderivati, sostituisce i set di infusione?________ |
| **4. Disinfetta i connettori senza ago posti sui set, prima di accedere alla porta d’infusione?**   mai **(andare alla domanda 5)**  raramente  talvolta  spesso  sempre   - 1. Quale agente antisettico utilizza?  clorexidina al 2%  iodopovidone  altro ________________ |
| **5.** **Lava i lumi del CVC dopo la somministrazione della terapia?**   mai **(andare alla domanda E1)**  raramente  talvolta  spesso  sempre  **5.1** Che cosa utilizza? **________________** |

1. **INFORMAZIONI**

**Le porgo alcune domande per conoscere le fonti e il bisogno di informazioni sulle batteriemie correlate al CVC.**

**E1.** Da quali delle seguenti fonti acquisisce informazioni in tema di batteriemie correlate ai CVC?

□ Nessuna □ Linee Guida □ Convegni/Corsi □ Colleghi □ Riviste scientifiche □ Internet □ Associazioni professionali

**E2.** Ritiene di aver bisogno di ulteriori informazioni sulle batteriemie correlate al CVC?  No  Si
