# Supplementary material for: Knowledge, attitudes, and practice on the prevention of central line-associated bloodstream infections among nurses in oncological care: A cross-sectional study in an area of southern Italy
Source: PLoS One. 2017 Jun 30;12(6):e0180473. doi: 10.1371/journal.pone.0180473 (PMC5493401; doi:10.1371/journal.pone.0180473)
Supplement: S2 Questionnaire — (DOC) [file pone.0180473.s002.doc]

**QUESTIONNAIRE**

1. **SOCIO-DEMOGRAPHIC AND PROFESSIONAL CHARACTERISTICS**

**I would like to gather information about your socio-demographic and professional characteristics.**

**A1**. What is your gender?  Male  Female **A2**. How old were you on your last birthday? **_______**

**A3**. Which is your highest educational qualification?

 Regional Diploma in nursing  University Diploma/Degree in nursing

**A4.** How many years have you been working in an oncology unit? **________**

**A5**. What is your professional role?  Ordinary nurse  Head-nurse/Nurses Coordinator

**A6**. In which unit do you work?  Ward  Outpatient Chemotherapy  Other**________________**

1. **KNOWLEDGE**

**I would like to explore your knowledge regarding central line-associated bloodstream infections (CLABSIs)**

| *Clinical practice guidelines are statements that include recommendations intended to optimize patient care that are informed by a systematic review of evidence and an assessment of the benefits and harms of alternative care options in order to assist health-care workers and patients* |
| --- |

**B1.** The Guidelines are needed in order to **(more than one answer is allowed)**:

**1.1**  reduce inappropriate variation in practice **1.2**  make decisions based on the best scientific evidence

**1.3**  reduce the risks for the patients **1.4**  provide appropriate health-care practice **1.5**  promote efficient use of resources

| **B2. Which are the recommended preventative measures for the CLABSIs?** | Yes | No | Do not know |
| --- | --- | --- | --- |
| **2.1** Flush the lumen with saline after the administration of medication or fluid |  |  |  |
| **2.2** Use sterile gauze or sterile transparent semi-permeable dressing to cover the catheter site |  |  |  |
| **2.3** Disinfect the needleless connectors before administer medication or fluid |  |  |  |
| **2.4** Replace catheter site dressing every 7 days for sterile semipermeable dressing or if the dressing becomes visibly soiled or loosened |  |  |  |
| **2.5** Use topical antibiotic ointment on catheter insertion site |  |  |  |
| **2.6** Replace the IV administration sets every 72 hours |  |  |  |
| **2.7** Disinfect the catheter insertion site with hydrogen peroxide |  |  |  |
| **2.8** Routine use of anticoagulants solutions |  |  |  |

1. **ATTITUDES**

**I would like to know your attitudes towards CLABSIs. Answer the following questions as truthfully as possible**

C1. How useful do you think the CLABSIs prevention guidelines are, on a 1 to 10 scale where 1 indicates useless and 10 indicates very useful

1 2 3 4 5 6 7 8 9 10

C2. How do you perceive your risk of transmitting a CLABSI when handling the CVC, on a 1 to 10 scale where 1 indicates no risk and 10 indicates very high risk

1 2 3 4 5 6 7 8 9 10

**C3.** How would you rate the utility of hand hygiene before and after the replacement of the dressing to reduce CLABSIs, on a 1 to 10 scale where 1 indicates useless and 10 indicates very useful?

1 2 3 4 5 6 7 8 9 10

| **For each statement answer whether you agree, uncertain, disagree for the CLABSIs prevention** | **Agree** | **Uncertain** | **Disagree** |
| --- | --- | --- | --- |
| **C.4** Monitor the catheter sites visually or by palpation through an intact dressing on a regular basis |  |  |  |
| **C.5** Allow the antiseptic on the insertion site to dry before catheter insertion |  |  |  |
| **C.6** The use of gloves before infusion port access replaces the need for hand washing |  |  |  |

1. **BEHAVIORS**

**I would like to gather information about your behavior**

**D1**. **Which of the following actions do you perform in CVCs management?**

| 1. **Do you change the dressing on the CVC insertion site?**   Yes No **(go to question 2)**    1. How often do you wash your hands before changing the CVC site dressing?    never **(go to question 1.3)**  rarely  sometimes  often  always  **1.2** What do you use to wash your hands?  antiseptic  soap  other **___________**  **1.2.1** How long do you wash your hands? **_________________**  **1.3** How often do you wear gloves before changing the CVC site dressing?   never **(go to question 1.4)**  rarely  sometimes  often  always  **1.3.1** What kind of gloves do you use?  non-sterile   initially non-sterile for removing dressing, then sterile for change the dressing   non-sterile with “no touch” technique   sterile  **1.4** After how many days do you replace the dressing? ________  **1.5.** What do you use for skin antiseptic preparation?  chlorhexidine 2%  povidone iodine  other ___________  **1.6**. Do you clean the CVC site before disinfection?  No **(go to question 2)**   Yes, with:  normal saline  other ________________  **1.7** Do you allow the antiseptic to dry before proceeding?  No **(go to question 2)**  Yes **1.7.1** For how long? ______ |
| --- |
| 1. **Do you perform the disinfection of the CVC access port before administer therapy?**    never **(go to question 3)**  rarely  sometimes  often  always  **2.1** What do you use?  chlorhexidine 2%  povidone iodine  other ________________  **2.2** Do you allow the antiseptic to dry before proceeding? No  **(go to question 3)**  Yes **2.2.1** For how long? ______ |
| **3. Do you replace the intravenous (IV) administration sets?**  Yes  No **(go to question 4)**  **3.1** Do you wash your hands before the replacement of administration sets?   never  rarely  sometimes  often  always  **3.2** Do you wear gloves before the replacement of administration sets?   never  rarely  sometimes  often  always  **3.3** How many hours after the infusion with lipid emulsions, do you replace the administration sets? **_________**  **3.4** How many hours after the infusion with blood and blood products, do you replace the administration sets?________ |
| **4. Do you disinfect the IV access port needleless connectors before accessing or manipulation?**   never **(go to question 5)**  rarely  sometimes  often  always   - 1. What antiseptic agent do you use?  chlorhexidine 2%  povidone iodine  other ________________ |
| **5.** **Do you flush the CVC lumens after IV therapy?**   never **(go to question E1)**  rarely  sometimes  often  always  **5.1** What do you use? **________________** |

1. **INFORMATION**

**I am going to ask you some questions to know the sources and need of information about CLABSIs.**

**E1.** From which of the following sources did you receive information about CLABSIs?

□ None □ Guidelines □ Workshops/Courses □ Colleagues □ Scientific journals □ Internet □ Professional organization

**E2.** Do you feel you need more information about CLABSIs?  No  Yes
